# Public support for more stringent vaccine policies increases with vaccine effectiveness

**DOI:** 10.1038/s41598-024-51654-y

**Published:** 2024-01-19

**Authors:** Richard Koenig, Manu Manthri Savani, Blake Lee-Whiting, John McAndrews, Sanchayan Banerjee, Andrew Hunter, Peter John, Peter John Loewen, Brendan Nyhan

**Affiliations:** 1 Department of Political Economy, King’s College London, London, WC2R 2LS UK; 2 Department of Social and Political Sciences, Brunel University London, London, UB8 3PH UK; 3 Munk School of Global Affairs and Public Policy, University of Toronto, Toronto, M5S 3K9 Canada; 4 Department of Political Science, University of Toronto, Toronto, M5S 3G3 Canada; 5 Institute for Environmental Studies (IVM), Vrije Universiteit Amsterdam, Amsterdam, 1081 HV The Netherlands; 6 Department of Government, Dartmouth College, Hanover, 03755 USA; 7 Digital Society Lab, McMaster University, Hamilton, L8S 4L8 Canada

**Keywords:** Health policy, Public health

## Abstract

Under what conditions do citizens support coercive public policies? Although recent research suggests that people prefer policies that preserve freedom of choice, such as behavioural nudges, many citizens accepted stringent policy interventions like fines and mandates to promote vaccination during the COVID-19 pandemic—a pattern that may be linked to the unusually high effectiveness of COVID-19 vaccines. We conducted a large online survey experiment (N = 42,417) in the Group of Seven (G-7) countries investigating the relationship between a policy’s effectiveness and public support for stringent policies. Our results indicate that public support for stringent vaccination policies increases as vaccine effectiveness increases, but at a modest scale. This relationship flattens at higher levels of vaccine effectiveness. These results suggest that intervention effectiveness can be a significant predictor of support for coercive policies but only up to some threshold of effectiveness.

## Introduction

The COVID-19 pandemic saw governments impose substantial restrictions on citizens. How do citizens respond to government use of coercive public policy measures? In many democratic countries, the public generally complied with interventions to promote vaccination^1^ and curtail the transmission of COVID-19, particularly early in the pandemic^2–4^. Efforts to promote vaccination and boosters enable us to learn about what kinds of state action citizens in established democracies are willing to support, and under what conditions. Using data from experiments in seven democracies, we find that citizens’ support for stringent booster policies depends on the effectiveness of boosters. Support for more stringent policies increases with booster effectiveness up to a point; beyond this threshold, public support for stringent policies does not measurably change. In general terms, we find that citizens appear to take policy effectiveness into account when considering whether to support stringent policies.

Public policy literature organizes the levers of policy action available to governments in various typologies and frameworks according to the nature of government intervention^5–9^. Previous work distinguishes between policy instruments that rely more on persuasion to facilitate behaviour change, and policies which exert greater pressure to comply (i.e., “carrots” and “sticks”^10^). Less stringent policies raise awareness, share information, and appeal to social norms to promote behaviour change. More stringent policies mandate action through laws and regulations and/or overtly raise the costs of non-compliance through financial incentives or disincentives. These approaches can thus be understood to vary on a continuum of “policy stringency”^11^. In the context of COVID-19, more stringent policies might include measures like limiting citizen travel and mobility, issuing curfews, closing shopping centers, or limiting employment opportunities for the unvaccinated. Stringency of COVID-19 containment measures has been operationalised through initiatives such as the COVID-19 Policy Stringency Index^12^.

In general, people express a preference for less stringent interventions in domains such as public health, savings, and the environment^13,14^. Citizens generally prefer being offered freedom to choose rather than being mandated to comply^15^. However, many people largely complied with more stringent policies that were put in place to promote vaccination during the COVID-19 pandemic such as financial incentives, travel restrictions, and employer mandates^16^. Such a result should therefore be seen as surprising, and has prompted new research on the trade-offs of personal freedoms for public health gains^17^. The compliance we observed with such coercive policies could be explained in part by the effectiveness of the COVID-19 vaccines—a factor potentially affecting public support for coercive policies that has not been adequately explored in prior research^18^.

It is important to understand the conditions under which citizens will support coercive policies. In the context of vaccines, there are two broad possibilities. Vaccines require a certain level of uptake to ensure population-level benefits. These required levels of uptake will increase when vaccines are less effective. Are citizens more supportive of coercive policies when vaccines are less effective, understanding that overcoming individual-level reluctance to take less effective vaccines will be required to achieve broad uptake? Or, are they more supportive of coercive policies when vaccines are more effective because more uptake will provide both individual- and population-level protection?

To explore the relationship between vaccine effectiveness and citizen support for coercive policies, we conducted two cross-national survey experiments in seven countries. We obtained nationally representative samples in seven democratic and high-income countries (Canada, France, Germany, Italy, Japan, UK, and US) from a commercial sample provider using quotas for age, gender, education, and sub-national region. Each survey contained a between-subjects experiment asking respondents about a hypothetical future scenario in which the government encouraged individuals to get a new booster vaccine for a new COVID-19 variant. Study 1 (N = 24,303) was fielded from 27 January to 26 February 2022 and Study 2 (N = 18,114) was fielded from 4 March to 12 May 2022. Information was given to respondents about the effectiveness of the hypothetical booster. Respondents were randomly assigned to receive different information vignettes. The experiment in Study 1 described the booster’s effectiveness numerically as 50%, 60%, 70%, 80%, or 90%. Building on this approach, the experiment in Study 2 tests an alternative framing by presenting effectiveness in relative terms— “less effective,” “as effective,” or “more effective” than previous COVID-19 vaccines. After receiving information about the effectiveness of the vaccine, respondents were then asked whether they would support a series of policy interventions the government could use to encourage greater uptake that ranged from less stringent (freely available boosters) to more stringent (restrictions on accessing public spaces for the unvaccinated, employer mandates for the booster, fines for the unvaccinated, and tax breaks for the vaccinated). The experimental design allows us to draw causal inference of the effect of the information received on support for stringent policies.

Across both studies, we found that the public is indeed more willing to support stringent booster policies when the booster’s effectiveness is greater. However, we observe diminishing marginal returns; there is no significant increase in support for more stringent policies when effectiveness is above 70% (in Study 1) or “more effective” than previous vaccines (in Study 2). We conclude the public is more willing to support stringent measures as the effectiveness of a hypothetical booster increases, but not measurably so after a certain point—a finding that has important implications for the design of vaccine promotion policies and information campaigns.

## Study 1

### Method

We administered the first preregistered online survey experiment in Canada, France, Germany, Italy, Japan, United Kingdom, and the United States from 27 January to 26 February 2022 (following ethical approval by King’s College London’s research ethics committee; all methods were performed in accordance with the relevant guidelines and regulations). The experiment was embedded in a survey designed on Qualtrics and fielded to national samples supplied by Dynata—an international sample provider—using quotas for age, gender, education, and subnational region. Respondents were compensated at rates set by the sample provider. The survey was written in English and professionally translated into French, German, Italian, and Japanese; the translations were also cross-validated by a different set of native speakers affiliated with the research team. Informed consent was sought and given by all participants. All survey materials are publicly available. The experiment was part of a larger multi-country study. We preregistered our hypothesis that the public will support more stringent policy measures as booster effectiveness increases; the primary outcome variable (support for proposed policy measures); treatment vignettes; planned sample size; and statistical model specifications (https://osf.io/6mkwg/).

### Experimental procedure

Using a between-subjects design, we randomized respondents into one of five treatment conditions. In each condition, participants were presented with a hypothetical scenario set in October 2022 in which a new booster is developed to protect against a new variant of COVID-19. The participants were then informed about the effectiveness of the booster in preventing infection. Participants were randomly assigned to receive different information about the level of protection (50%, 60%, 70%, 80%, 90%) and were then asked whether they would support a series of policies aimed at encouraging the take-up of the booster (see Table 1). By comparing participants’ responses across the randomly assigned conditions, this experimental design allows us to estimate the causal effect of the information—i.e., the effectiveness of a hypothetical booster—on support for stringent policies. Survey experiments are used extensively to estimate causal effects on individuals’ attitudes and self-reported behaviours, including in the context of COVID-19^19–21^.

**Table 1:** Study 1 experimental instrument.

Imagine the following scenario: In October of 2022, a new variant emerges which, like Omicron, is highly contagious. New vaccine boosters are developed. These boosters provide **[50% / 60% / 70% / 80% / 90%]** protection against infection from the new variant. The government in your country would like individuals to take this booster shot. Which of the following policies would you support?	
**Policy 1**: No one should be forced to take the booster, but it should be available to anyone who wants it.	Yes / No / Unsure
**Policy 2**: Those who do not take the booster should be stopped from entering any indoor public spaces (e.g. restaurants, entertainment venues) and/or using public transport.	Yes / No / Unsure
**Policy 3**: Employers should require their employees to get the booster.	Yes / No / Unsure
**Policy 4**: Those who do not take the booster should be fined by the government.	Yes / No / Unsure

### Analysis

Per our pre-registration, we pool observations across countries. The final sample for analysis was 24,303 participants (50% effectiveness condition N = 4785, 19.7% of sample; 60% condition N = 4999, 20.6%; 70% condition N = 4947, 20.4%; 80% condition N = 4762, 19.6%; 90% condition N = 4810, 19.8%). We excluded observations for the following reasons: multiple responses from the same survey respondent ID provided by Dynata, not living in the country in which the survey was administered, incomplete responses, surpluses in sample quotas, responses flagged by Qualtrics as “spam”, and failure to pass an attention screen. In addition, we deviate from the preregistration to remove 27 observations due to a technical issue (specifically, due to an unanticipated failure to load JavaScript programming required for randomizing the experimental instrument). For summary statistics by treatment group, see Appendix Table A1.

Our outcome variable is an index of support for stringent booster policies ranging from 0 to 4 where a higher score indicates greater support for more stringent booster policies (hereafter the booster policy support index). To calculate the booster policy support index, respondents were given one point for each policy they supported with the exception of the first policy. Here respondents were given a point for saying “No” to “No one should be forced to take the booster”, because opposition to Policy 1 indicates an openness to greater government intervention. (The first policy was inadvertently omitted from the preregistration; we deviate to reverse-code the first item as part of the list of four items.) Mean support for each policy and for the index are reported by condition in Table 2.

**Table 2:** Support for each booster policy by treatment condition (Study 1).

Experimental group	Policy 1	Policy 2	Policy 3	Policy 4	Booster policy support index
50%	0.18	0.45	0.45	0.27	1.35
	(0.006)	(0.007)	(0.007)	(0.006)	(0.007)
60%	0.18	0.45	0.46	0.27	1.36
	(0.005)	(0.007)	(0.007)	(0.006)	(0.006)
70%	0.20	0.47	0.47	0.29	1.43
	(0.006)	(0.007)	(0.007)	(0.006)	(0.007)
80%	0.19	0.47	0.48	0.29	1.43
	(0.006)	(0.007)	(0.007)	(0.007)	(0.007)
90%	0.19	0.48	0.47	0.28	1.41
	(0.006)	(0.007)	(0.007)	(0.006)	(0.007)
Overall mean support for policy	0.19 (0.006)	0.47 (0.007)	0.47 (0.007)	0.28 (0.006)	1.40 (0.007)

Mean policy support by treatment with standard errors of means in parentheses. Means represent the proportion of people who said ‘yes’ to Policies 2, 3, and 4 (the stringent policies); and who said ‘no’ in response to Policy 1. Overall mean support for each individual policy is reported in the final row. Policy support is summed by experimental group to generate the booster policy support index.

As an initial test of our hypothesis, we estimated our first preregistered model in which the treatment is modeled as a continuous independent variable. We estimate intent-to-treat (ITT) effects using OLS with robust standard errors and randomization-*t p*-values to account for multiple comparisons. The model includes country fixed effects (FE) with additional covariates selected from a preregistered list using a lasso procedure to increase the precision of our treatment effect estimates^22^. The results are reported in Appendix Table A2 (Column 1). Consistent with our expectations, we find that greater booster effectiveness increases policy support, albeit modestly: a ten-percentage point increase in booster effectiveness increases the booster policy support index by 0.017 (*p* = .002) on a scale from 0 to 4, which translates to an increase of approximately 0.012 standard deviations—a small treatment effect, but in the expected direction.

**Figure 1:**
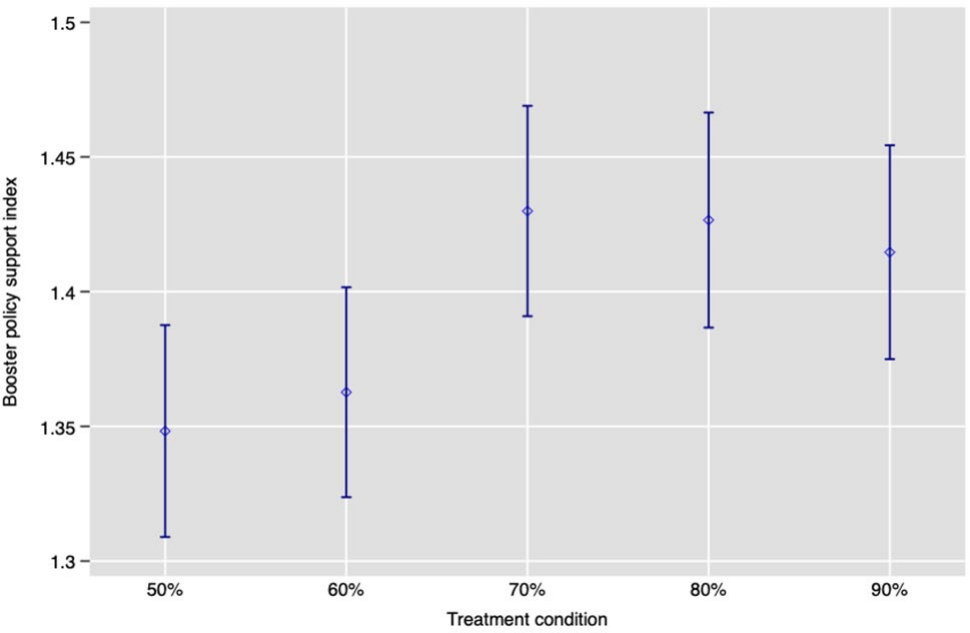
Mean booster policy stringency index by treatment condition with 95% CIs (Study 1). Support for more stringent policies is greater when vaccine effectiveness is greater, but support plateaus beyond 70% vaccine effectiveness.

For robustness, we also investigated whether the treatment effect was linear or whether, after a certain point, the positive effect of booster effectiveness begins to level off. To explore this possibility, Figure 1 reports the mean of the booster policy support index in each of the five randomized treatment conditions. The figure provides clear evidence of a non-linear effect. In particular, we find notably greater support in the 70% condition relative to the 50% condition, but little discernible increase in support beyond 70% booster effectiveness.

To assess this non-linearity more formally, we estimated our second preregistered model in which the treatment is modelled as a 5-category variable using the 50% condition as the baseline reference category (see Table 3). Per our preregistration, we again use OLS regression to estimate intent-to-treat effects with robust standard errors, fixed effects, and covariates selected using a lasso procedure^22^. Consistent with Figure 1, Table 2 underscores the notable levelling-off of the treatment effect. Moving from 50% effectiveness to 70% effectiveness leads to a 0.061 increase in the booster policy support index, which translates to 0.044 standard deviations. Moving from the same baseline of 50% effectiveness to 90% effectiveness (i.e., double the increase in booster effectiveness) also yields the same 0.061 increase in the index. In Appendix Table A3, we report the ITT effects separately for each policy; we find statistically significant evidence of treatment effects for Policies 1 and 2, but not for Policies 3 and 4. We further test whether our findings are robust to an alternative construction of our index that estimates latent policy support (see Appendix Table A7), and confirm that the same positive relationship is found between booster effectiveness and support for stringent policy.

**Table 3:** ITT effect when booster effectiveness is modelled as a categorical variable (Study 1).

	Booster policy support index (0–4) Coefficient (SE)
60% effective	0.019 (0.025)
70% effective	0.061* (0.025)
80% effective	0.068* (0.025)
90% effective	0.061* (0.025)
Constant	−0.138*** (0.04)
Controls	✓
Country FE	✓
N	24,005

OLS estimates with heteroskedasticity-robust standard errors in parentheses. We use randomization-*t p*-values^20^ to account for multiple comparisons: *** *p*<0.005, ** *p*<0.01, * *p*<0.05. 50% effective is the baseline category. Controls selected by lasso linear regression specification^22^ to increase the precision of our treatment effect estimates, from our preregistered list of covariates: age, gender, education, parental status, town/city type, religious beliefs, political left-right scale, risk preference, previous COVID-19 infection (self), previous COVID-19 infection (household), vaccination status, booster status, and trust in vaccines (binary). The data are unweighted. We were unable to include 298 observations in the regression analysis due to missing values in the lasso controls.

## Study 2

### Method

Study 2 was fielded from 4 March to 12 May 2022 with new samples from the G-7 countries, following the same sampling and translation procedures as in Study 1 and separately preregistered (https://osf.io/f86vz/). Ethical approval was granted by King’s College London’s research ethics committee; all methods were performed in accordance with the relevant guidelines and regulations. Informed consent was sought and given by all participants.

### Experimental procedure

Study 2 differed from Study 1 in two key respects. First, we sought to address the concern that participants in Study 1 might not understand the effectiveness statistics presented. We therefore expressed the randomized efficacy information provided to participants in Study 2 in terms relative to prior COVID-19 boosters: “less effective,” “as effective,” or “more effective” (instead of 50–90%). Second, although the outcome variable remained a booster policy support index, participants were now presented with seven instead of four policies and the response options of “I would support this” and “I would oppose this” instead of “Yes”, “No” and “Unsure”. The aim was to capture greater variation in the stringency and costs imposed by policies, which were designed to mirror real world interventions^16^. Table 4 displays the survey instrument.

**Table 4:** Study 2 experimental instrument.

Next, we’d like you to imagine a new hypothetical scenario: A new and highly contagious variant of COVID-19 emerges several months from now. A new booster has been developed to combat this new variant. This new booster is safe and recommended for everyone regardless of whether they previously had any COVID-19 vaccinations. This new booster is **[“less” / “as” / “more”]** effective at preventing COVID-19 infection than previous vaccines. The government would like people to get this new booster. In this scenario, which of the following policies would you support?	
Policy 1: The government makes this new booster freely available to all eligible adults.	I would support / I would oppose this
Policy 2: The government promotes this new booster using advertisements.	I would support / I would oppose this
Policy 3: The government sends text messages to eligible adults reminding them that this new booster is available.	I would support / I would oppose this
Policy 4: The government gives a tax break to eligible adults who get this new booster.	I would support / I would oppose this
Policy 5: The government allows employers to require their eligible employees to get this new booster.	I would support / I would oppose this
Policy 6: The government requires eligible adults to show proof that they got this new booster before they can enter certain indoor places such as restaurants, gyms, and theatres.	I would support / I would oppose this
Policy 7: The government fines eligible adults who refuse this new booster.	I would support / I would oppose this

### Analysis

After excluding participants using the same criteria specified in Study 1, the final analysis sample was 18,114 participants (less effective condition N = 6084, 33.6% of sample; equally effective N = 6050, 33.4%; more effective N = 5980, 33.0%). Appendix Table A4 presents summary statistics by treatment group.

The booster policy support index is now an eight-point scale (0–7) counting how many policies each respondent supports. To calculate the booster policy support index, respondents were given one point for each policy they supported. A higher score again indicates greater support for stringent booster policy. Table 5 presents mean scores for each policy by treatment group.

**Table 5:** Support for each booster policy by treatment condition (Study 2).

Experimental group	Policy 1	Policy 2	Policy 3	Policy 4	Policy 5	Policy 6	Policy 7	Booster policy support index
Less effective	0.81	0.69	0.69	0.59	0.54	0.57	0.34	4.23
	(0.005)	(0.006)	(0.006)	(0.006)	(0.006)	(0.006)	(0.006)	(0.006)
Equally effective	0.84	0.74	0.74	0.63	0.59	0.62	0.38	4.54
	(0.005)	(0.006)	(0.006)	(0.006)	(0.006)	(0.006)	(0.006)	(0.006)
More effective	0.85	0.75	0.73	0.64	0.59	0.63	0.37	4.56
	(0.005)	(0.006)	(0.006)	(0.006)	(0.006)	(0.006)	(0.006)	(0.006)
Overall mean support for policy	0.83 (0.005)	0.73 (0.006)	0.72 (0.006)	0.62 (0.006)	0.57 (0.006)	0.61 (0.006)	0.36 (0.006)	4.44 (0.006)

Mean policy support by treatment with standard errors of means in parentheses. Means for policies 1 to 7 represent the proportion of people who said ‘yes’ to Policies 2–7 (the stringent policies); and who said ‘no’ in response to Policy 1. Overall mean support for each individual policy is reported in the final row. Policy support is summed by experimental group to generate the booster policy support index.

To formally test our hypothesis, we first use the same preregistered method as in Study 1 (ITT estimates using OLS with the same lasso covariate selection procedure and country FEs with robust SEs). The results are presented in Table A2 (Column 2). Consistent with our expectations, we find that greater booster effectiveness modestly increases support for more stringent policies: a unit increase in booster effectiveness (going from “less effective” to “as effective” or from “as effective” to “more effective”) increases the booster policy support index by 0.156 (*p* < *.*001) on a 0 to 7 scale, which translates to an increase of approximately 0.068 standard deviations.

**Figure 2:**
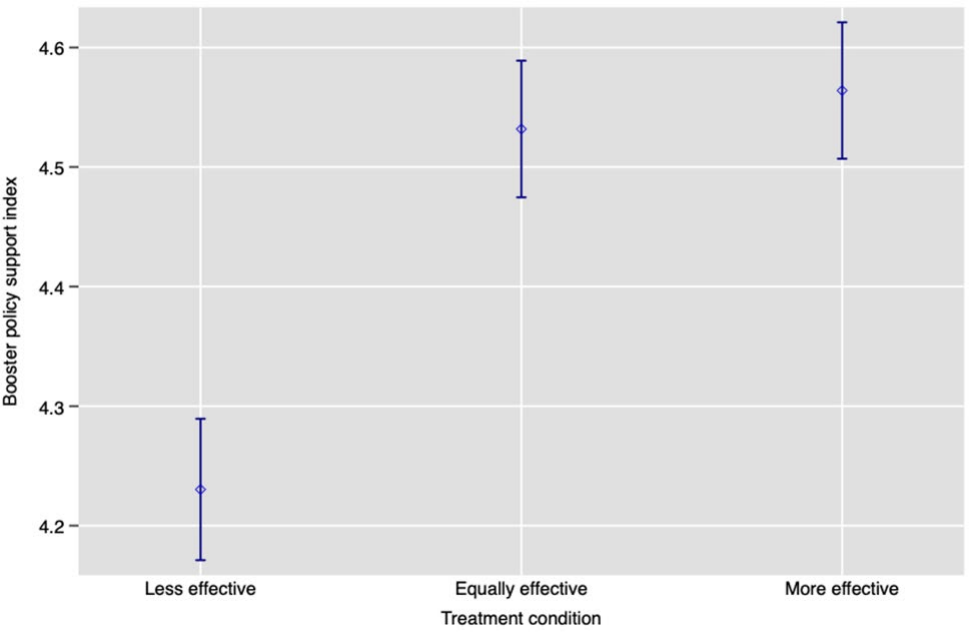
Mean booster policy stringency index by treatment condition with 95% CIs (Study 2). Support for more stringent policies is greater when the booster is presented as “equally” or “more effective”.

We also investigate the linearity of the treatment effect in Study 2. Initial evidence of non-linearity is provided in Figure 2, which shows support for stringent booster policies increases substantially when the booster is “equally effective” to previous vaccines rather than “less effective”. However, support does not increase discernibly when the booster is instead described as “more effective” than previous vaccines.

We then investigated this non-linearity more formally by regressing the 8-point index on the 3-category treatment, using the “less effective” condition as the baseline. Here, we deviate from the preregistered model (whose results are presented in Appendix Table A5) in which the baseline category was “equally effective”. We instead chose “less effective” as a baseline to better match the model from Study 1 where the least effective booster (50% effective) was the baseline, making results from the two studies more comparable. Table 6 presents the results from this model. Moving from a “less effective” booster to an “equally effective” booster leads to a 0.265 (*p* < *.*001) increase in the booster policy support index, which translates to 0.116 standard deviations. By contrast, moving from a “less effective” booster to one that is “more effective” yields a similar 0.309 (*p* < *.*001) increase in the booster policy support index (0.135 standard deviations). The difference between the “equally” and “more effective” conditions is not statistically significant (*p* > *.*05). In Appendix Table A6, we report the ITT effects separately for each policy; we find statistically significant evidence of treatment effects for each of the seven policies. As a further robustness check on our policy support index, we compare our findings with an alternative index based on latent support for stringent policy (see Appendix Table A8) and recover the same results.

**Table 6:** ITT effect when booster effectiveness is modelled as a categorical variable (Study 2).

	Booster policy support index (0–7) Coefficient (SE)
Equally effective	0.265*** (0.034)
More effective	0.309*** (0.034)
Constant	1.105*** (0.092)
Controls	✓
Country FE	✓
N	17,306

OLS estimates with heteroskedasticity-robust standard errors in parentheses. We use randomization-*t p*-values^23^ to account for multiple comparisons: *** *p*<0.005, ** *p*<0.01, * *p*<0.05. 50% effective is the baseline category. Controls selected by lasso linear regression specification^22^ to increase the precision of our treatment effect estimates, from our preregistered list of covariates: age, gender, education, parental status, town/city type, religious beliefs, political left-right scale, risk preference, previous COVID-19 infection (self), previous COVID-19 infection (household), vaccination status, booster status, and trust in vaccines (binary). The data are unweighted. We were unable to include 808 observations in the regression analysis due to missing values in the lasso controls.

These results confirm the relationship between booster effectiveness and support for stringent policies. Support for policy stringency increases as vaccine effectiveness increases from a low baseline. However, an increase in booster effectiveness beyond the current COVID-19 vaccine status quo does not produce an equivalent increase in support for stringent policies, suggesting that intervention effectiveness does not enhance public support beyond a certain threshold.

## Discussion

Governments often wish to encourage citizens to engage in behaviours such as using public transit or receiving vaccines. They may similarly wish to discourage citizens from behaviors like smoking or driving under the influence of alcohol. When are citizens willing to support more stringent or coercive policies from governments? We contribute to scholarly debates on public attitudes towards more and less stringent policy instruments^18,24,25^, and how the content of vaccine promotion messages might influence vaccination intentions^26–28^. Our findings suggest citizens’ willingness to increase the restrictions placed on themselves and other citizens depends on the relative effectiveness of the behaviour being encouraged by the government.

Across two survey experiments with a combined N = 42,417 in the G-7 countries, we find that vaccine booster effectiveness increases public support for more stringent booster policies, but the effect is relatively small and depends on the performance of the booster. When participants are informed the booster has 70% rather than 50% effectiveness (Study 1) or that it is “equally effective” rather than “less effective” compared to current COVID-19 boosters (Study 2), we find higher support for stringent vaccination policies such as employer vaccine mandates, vaccine passes for public spaces, and fines for unvaccinated adults. These results conversely imply that reduced effectiveness decreases support for such policies.

We also found evidence of an apparent threshold effect, which was unanticipated. These findings suggest a notable asymmetry in preferences. Greater booster effectiveness increases support for stringent booster promotion policies primarily in the lower range of measured effectiveness; above a certain point, greater booster effectiveness does not yield similar marginal increases in support for stringent policies.

The reasons for this unanticipated finding are an important topic for future research. It is possible, for instance, that people reached a ceiling on the level of stringency that they were willing to accept; or that the trade-off between policy benefits and limits to individual freedom is less compelling as policy effectiveness increases above some level. Our findings have direct implications for policymakers. Though the effect sizes we observe are small, even modest effect sizes can have meaningful implications in the context of large-scale vaccination or booster programmes. The asymmetry in our findings is perhaps of particular relevance to policy makers, public health communicators, and public health information campaigns. If future vaccines are presented as, and/or are perceived to be, less effective than the initial vaccines, then we would expect citizens to be less accepting of many common vaccine promotion policies.

We conclude by noting several important avenues for future research. First, we focus on seven advanced economies with mature democracies, but different results might be obtained in middle- and low-income countries, or countries with different political systems or regime types. Second, these results might change over time as policies and public willingness to comply with them varies. Third, the results we observe here might vary in other (non-health) policy domains or for other vaccination programs such as influenza or vaccines for children. Future studies should therefore consider extensions in different countries, at different times, and in different domains. Such findings will not only provide important insight into the generalizability of our findings but help identify whether the reason for the asymmetry we observe is specific to COVID-19 vaccines and the pandemic or a more general feature of public opinion.

### Supplementary Information

Supplementary Information 1.

Supplementary Information 2.

Supplementary Information 3.

## Data Availability

The datasets used and analyzed during the current study are available from the corresponding author on reasonable request.
