# Supplementary figures and images for: Public support for more stringent vaccine policies increases with vaccine effectiveness
Source: Sci Rep. 2024 Jan 19;14:1748. doi: 10.1038/s41598-024-51654-y (PMC10798948; doi:10.1038/s41598-024-51654-y)

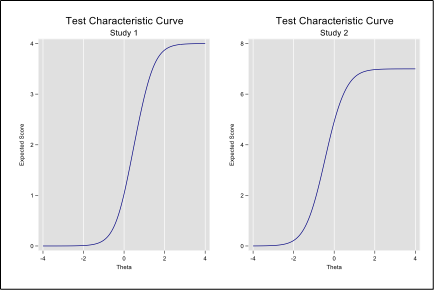

Supplement: Supplementary file 1 — Supplementary Information 1. [file 41598_2024_51654_MOESM1_ESM.png]

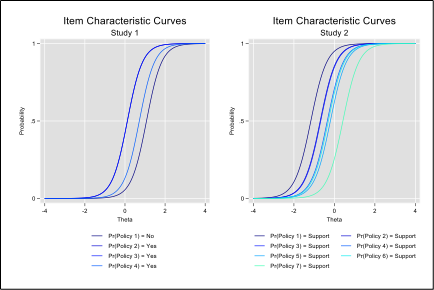

Supplement: Supplementary file 2 — Supplementary Information 2. [file 41598_2024_51654_MOESM2_ESM.png]
