# Supplementary material for: Public support for more stringent vaccine policies increases with vaccine effectiveness
Source: Sci Rep. 2024 Jan 19;14:1748. doi: 10.1038/s41598-024-51654-y (PMC10798948; doi:10.1038/s41598-024-51654-y)
Supplement: Supplementary file 3 — Supplementary Information 3. [file 41598_2024_51654_MOESM3_ESM.pdf]

Appendix

Table A1. Summary statistics by treatment condition (Study 1)

Effectiveness	Variable	N	Mean	SD
50%	Age	4785	47.35	16.35
	Male	4765	0.49	0.5
	University-educated	4785	0.29	0.46
60%	Age	4999	47.01	16.18
	Male	4975	0.48	0.5
	University-educated	4999	0.28	0.45
70%	Age	4947	47.46	16.56
	Male	4921	0.48	0.5
	University-educated	4947	0.3	0.46
80%	Age	4762	47.65	16.26
	Male	4745	0.48	0.5
	University-educated	4762	0.29	0.46
90%	Age	4810	46.91	16.34
	Male	4782	0.5	0.5
	University-educated	4810	0.31	0.46
Total	Age	24303	47.29	16.34
	Male	24188	0.49	0.5
	University-educated	24303	0.3	0.46

The data were collected from respondents living in seven democratic and high-income countries, namely Canada (N=3,470), France (N=3,485), Germany (N=3,485), Italy (N=3,487), Japan (N=3,421), UK (N=3,472), and US (N=3,483) from January to February 2022. University-educated refers to holding a Bachelor's or higher. As 115 respondents identified as non-binary, a different gender or preferred not to say, the N for Male is lower than the N for Age and University-educated.

Table A2. ITT effect when the treatment is modelled as a continuous variable (Study 1 in Column 1 and Study 2 in Column 2).

	Booster policy support index (0–4)	Booster policy support index (0–7)
Booster effectiveness (Study 1)	0.17***	
	(0.056)	
Booster effectiveness (Study 2)		0.156***
		(0.018)
Constant	-0.135***	1.047***
	(0.048)	(0.093)
Controls	✓	✓
Country FE	✓	✓
N	24,007	17,369

OLS estimates with heteroskedasticity-robust standard errors in parentheses. We use randomization-*t* *p*-values[20] to account for multiple comparisons: *** p<0.005, ** p<0.01, * p<0.05. Controls selected by lasso linear regression specification[19] to increase the precision of our treatment effect estimates from our pre-registered list of covariates: age, gender, education, parental status, town/city type, religious beliefs, political left-right scale, risk preference, previous COVID-19 infection (self), previous COVID-19 infection (household), vaccination status, booster status, and trust in vaccines (binary). The data are unweighted.

Table A3. ITT effect on support for individual policies when booster effectiveness is modelled as a categorical variable — "50% effective" as the baseline category (Study 1)

	Policy 1	Policy 2	Policy 3	Policy 4
60% effective	0.002	0.005	0.006	0.006
	(0.008)	(0.009)	(0.009)	(0.009)
70% effective	0.014	0.017	0.014	0.014
	(0.008)	(0.009)	(0.009)	(0.009)
80% effective	0.016*	0.016	0.017	0.016
	(0.0.008)	(0.009)	(0.009)	(0.009)
90% effective	0.009	0.027**	0.016	0.009
	(0.0.008)	(0.009)	(0.009)	(0.009)
Constant	0.099***	-0.037**	-0.119***	-0.105***
	(0.012)	(0.014)	(0.018)	(0.017)
Controls	✓	✓	✓	✓
Country FE	✓	✓	✓	✓
N	23,571	23,596	23,485	23,482

OLS estimates with heteroskedasticity-robust standard errors in parentheses. We use randomization-*t* *p*-values[20] to account for multiple comparisons: *** p<0.005, ** p<0.01, * p<0.05. Controls selected by lasso linear regression specification[19] to increase the precision of our treatment effect estimates from our pre-registered list of covariates: age, gender, education, parental status, town/city type, religious beliefs, political left-right scale, risk preference, age, gender, parental status, town/city type, religious beliefs, previous COVID-19 infection (self), previous COVID-19 infection (household), vaccination status, booster status, and trust in vaccines (binary). The data are unweighted.

Table A4. Summary statistics by treatment condition (Study 2)

Effectiveness	Variable	N	Mean	SD
Less effective	Age	6084	47.17	16.42
	Male	6054	0.48	0.5
	University-educated	6084	0.27	0.44
Equally effective	Age	6050	47.03	16.58
	Male	6023	0.47	0.5
	University-educated	6050	.27	0.45
More effective	Age	5980	47.22	16.37
	Male	5960	0.48	0.5
	University-educated	5980	0.27	0.44
Total	Age	18114	47.14	16.46
	Male	18037	0.48	0.5
	University-educated	18114	0.27	0.44

The data were collected from respondents living in seven democratic and high-income countries, namely Canada (N=2,566), France (N=2,623), Germany (N=2,568), Italy (N=2,565), Japan (N=2,674), UK (N=2,552), and US (N=2,566) from March to May 2022. University-educated refers to holding a Bachelor's or higher. As 77 respondents identified as non-binary, a different gender or preferred not to say, the N for Male is lower than the N for Age and University-educated.

Table A5. ITT effect when booster effectiveness is modelled as a categorical variable — "equally effective" as the baseline category (Study 2).

	Booster policy support index (0–7)
Less effective	-.265***
	(0.034)
More effective	0.044
	(0.032)
Constant	1.37***
	(0.093)
Controls	✓
Country FE	✓
N	17,306

OLS estimates with heteroskedasticity-robust standard errors in parentheses. We use randomisation-*t* *p*-values[20] to account for multiple comparisons: *** $p<0.005$, ** $p<0.01$, * $p<0.05$. Controls selected by lasso linear regression specification[19] to increase the precision of our treatment effect estimates from our pre-registered list of covariates: age, gender, education, parental status, town/city type, religious beliefs, political left-right scale, risk preference, previous COVID-19 infection (household), vaccination status, booster status, and trust in vaccines (binary). The data are unweighted.

Table A6. ITT effect on support for individual policies when booster effectiveness is modelled as a categorical variable — "less effective" as the baseline category (Study 2).

	Policy 1	Policy 2	Policy 3	Policy 4	Policy 5	Policy 6	Policy 7
Equally effective	0.018***	0.049***	0.041***	0.034***	0.043***	0.048***	0.032***
	(0.006)	(0.007)	(0.007)	(0.008)	(0.008)	(0.008)	(0.008)
More effective	0.033***	0.058***	0.038***	0.047***	0.042***	0.057***	0.032***
	(0.006)	(0.007)	(0.007)	(0.008)	(0.008)	(0.008)	(0.008)
Constant	0.494***	0.263***	0.237***	0.206***	0.016	0.002	-0.110***
	(0.018)	(0.017)	(0.018)	(0.02)	(0.021)	(0.021)	(0.021)
Controls	✓	✓	✓	✓	✓	✓	✓
Country FE	✓	✓	✓	✓	✓	✓	✓
N	17,406	17,472	17,990	17,409	17,407	17,392	17,396

OLS estimates with heteroskedasticity-robust standard errors in parentheses. We use randomization-*t* *p*-values[20] to account for multiple comparisons: *** $p<0.005$, ** $p<0.01$, * $p<0.05$. Controls selected by lasso linear regression specification[19] to increase the precision of our treatment effect estimates from our pre-registered list of covariates: age, gender, education, parental status, town/city type, religious beliefs, political left-right scale, risk preference, age, gender, parental status, town/city type, religious beliefs, previous COVID-19 infection (self), previous COVID-19 infection (household), vaccination status, booster status, and trust in vaccines (binary). The data are unweighted.

Robustness Checks on Policy Support Index

Below we present estimates based on a series of item response theory (IRT) one-parameter logistic models for Study 1 and Study 2 data respectively. These are based on the four policy items in Study 1, and the seven policy items in Study 2. Responses to each item are coded 1 if they are associated with higher levels of policy stringency, and 0 otherwise. To ensure the estimates derived from this model are directly comparable to that of main analysis, we performed a listwise deletion of observations for which at least one policy stringency item was not answered. Predictions from these models are used to generate a measure of latent attitudes to policy stringency (theta), which in turn provides an alternative weighting of the various items associated with policy stringency. The scale of this alternative weighting can be interpreted in terms of standard deviations from the mean of the latent attitude.

The Test Characteristic Curve links values of the latent attitude (x-axis) to the summative score of survey responses on stringent policy items.

Figure A1. Test Characteristic Curves (Study 1 and Study 2)

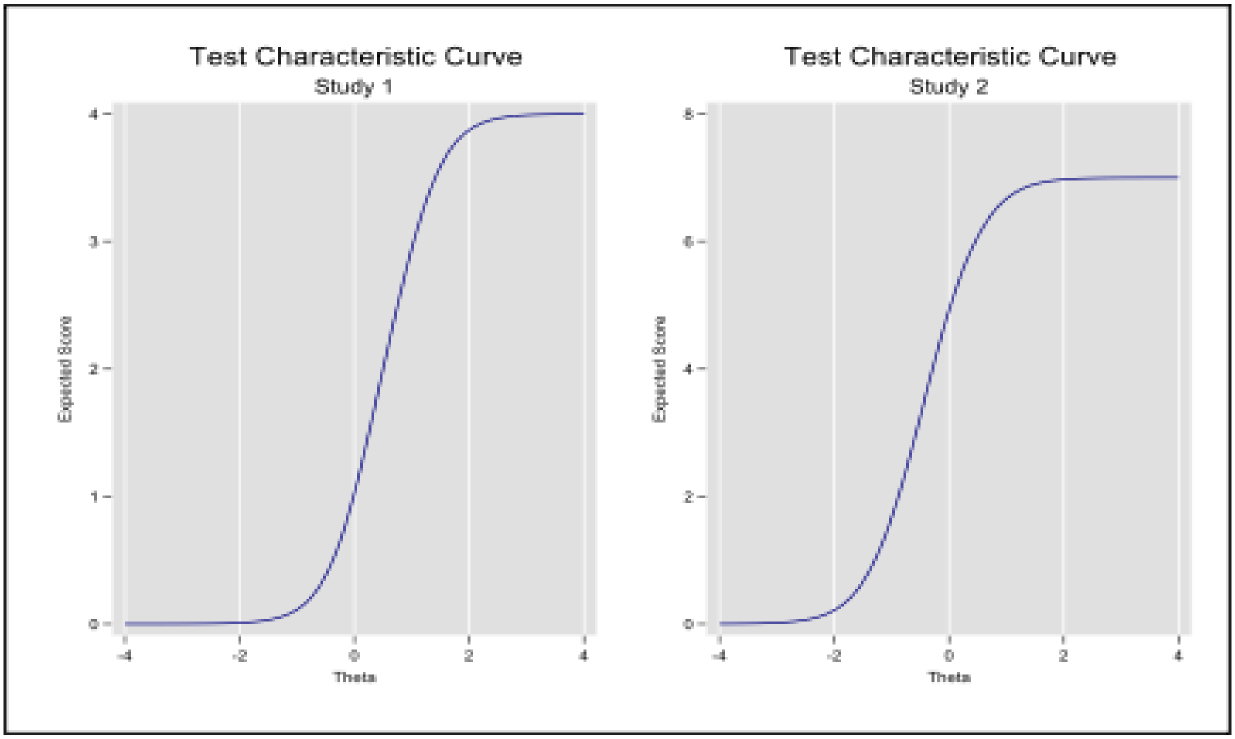

The Item Characteristic Curve shows the probability of answering each item with the outcome positively associated with policy stringency for varying levels of theta. The further to the right a particular curve, the higher the level of discrimination associated with the associated item. In other words, the further to the right a particular curve, the greater the "difficulty" of the associated item. This in turn means, positive responses to items with higher levels of discrimination are more indicative of stringent attitudes as a whole.

Figure A2. Item Characteristic Curves (Study 1 and Study 2)

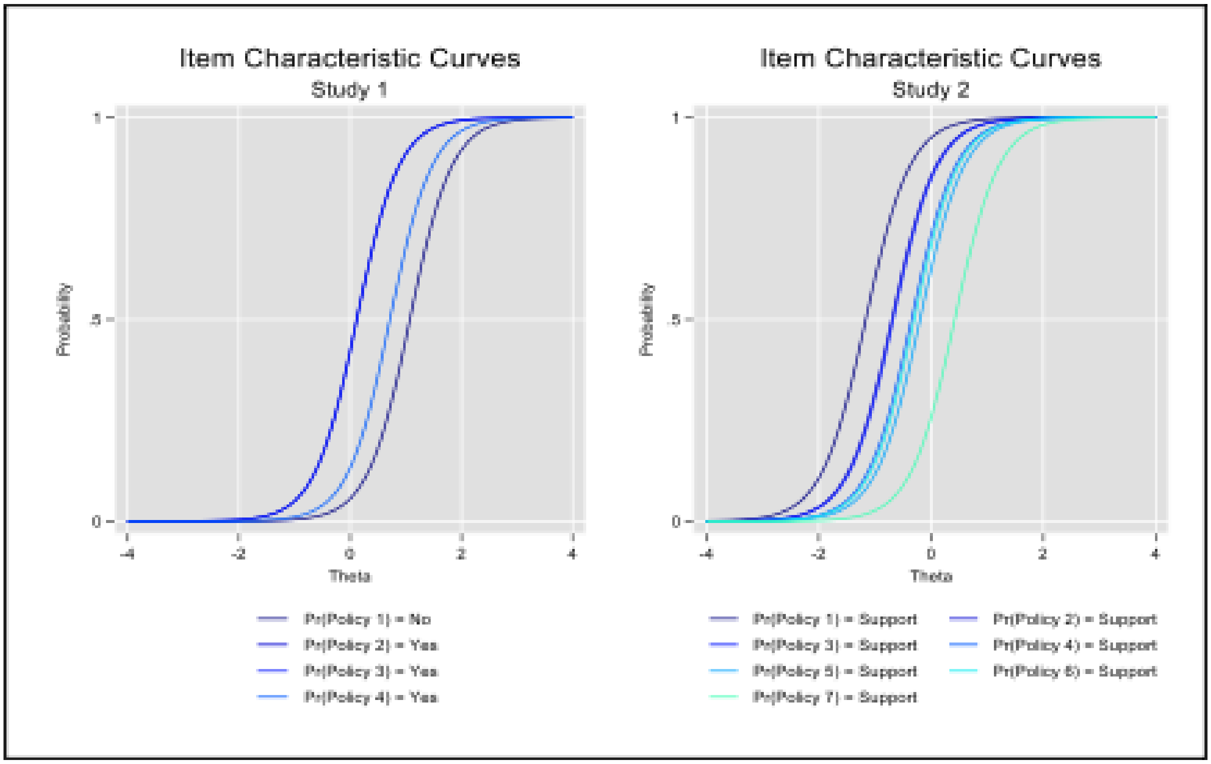

Using these weightings, we create an alternative index of support for stringent policy. The following tables replicate our main analyses – Table 3 for Study 1 and Table 6 for Study 2 – using the respective measures of policy support generated by the two IRT models described above. For a direct comparison with the results derived from the summative measure of stringent attitudes, we provide the results in Tables A7 and A8 rescaled in terms of standard deviations from the mean. The comparison demonstrates the results are robust to an alternative weighting of policy stringency items.

Table A7. Study 1 Replication of main results with IRT index of latent support for stringent policy

Treatment group	Original index<br>Std Dev from mean<br>(1)	IRT index<br>Std Dev from mean<br>(2)
60%	0.01	0.01
	(0.02)	(0.01)
70%	0.04*	0.04*
	(0.02)	(0.01)
80%	0.05**	0.04**
	(0.02)	(0.01)
90%	0.04*	0.04*
	(0.02)	(0.01)
N	24,005	24,005
Controls	Yes	Yes

Column 1 presents the results of our original and pre-registered analysis (using the summative index) in terms of standard deviations from the mean. Column 2 presents the results of IRT analysis (using the latent variable index) in terms of standard deviations from the mean. Model specification as in Table 3. *** $p<0.005$, ** $p<0.01$, * $p<0.05$.

Table A8. Study 2 IRT Replication of main results with IRT index of latent support for stringent policy

Treatment group	Original index<br>Std Dev from mean<br>(1)	IRT index<br>Std Dev from mean<br>(2)
Equally effective	0.12***	0.10***
	(0.01)	(0.01)
More effective	0.14***	0.12***
	(0.01)	(0.01)
N	17,306	17,306
Controls	Yes	Yes

Column 1 presents the results of our original and pre-registered analysis (using the summative index) in terms of standard deviations from the mean. Column 2 presents the results of IRT analysis (using the latent variable index) in terms of standard deviations from the mean. Model specification as in Table 6. *** $p<0.005$, ** $p<0.01$, * $p<0.05$.
